# Molecular Mechanisms of Efficacy Variation in Antivenoms: Insights from a Malayan Pit Viper (*Calloselasma rhodostoma*) Bite in Vietnam

**DOI:** 10.3390/tropicalmed10120331

**Published:** 2025-11-25

**Authors:** Thuan Quang Le, Nguyen Trung Nguyen, Yen Bao Pham, Minh Bao Vu, Nhan Thanh Le, Nhan Sy Pham Nguyen, Neil R. Balchan, Choo Hock Tan, Thai Huu Duong, Hoang Huy Nguyen, Tao Thien Nguyen

**Affiliations:** 1 Poison Control Center, Bach Mai Hospital, 78 Giai Phong Street, Phuong Mai Ward, Dong Da District, Hanoi 10000, Vietnam; 2 Falculty of Biology, VNU University of Science, 334 Nguyen Trai, Thanh Xuan, Hanoi 10000, Vietnam; 3 The 108 Military Central Hospital, 1 Tran Hung Dao, Hanoi 10000, Vietnam; 4 Department of Biology, Oklahoma State University, Stillwater, OK 74078, USA; 5 School of Medicine, College of Life Sciences and Medicine, National Tsing Hua University, Hsinchu 300044, Taiwan; 6 Department of Pharmacology, Faculty of Medicine, University Malaya, Kuala Lumpur 50603, Malaysia; 7 Institute of Vaccines and Medical Biologicals, Nha Trang 57106, Vietnam; 8 Institute of Biology, Vietnam Academy of Science and Technology, 18 Hoang Quoc Viet Road, Hanoi 10072, Vietnam

**Keywords:** antivenom, *Calloselasma rhodostoma*, envenomation, protein composition, snakebite, Vietnam

## Abstract

**Background:** Although antivenom is the standard treatment for snakebite envenoming, its efficacy may be impacted by geographic variation in venom composition, emphasizing the need for region-specific antivenom development. **Methods:** We report a case of snakebite envenoming, in which the patient was bitten on the hand by a captive Malayan pit viper (*Calloselasma rhodostoma*) with typical clinical manifestations following. Antivenom (produced in Thailand) was administered at 33 and 39 h post-bite. Venom from the causative individual snake was collected for compositional analysis via SDS-PAGE. Enzymatic activity of the venom was evaluated through the degradation of casein and phospholipid substrates, along with the assessment of enzymatic inhibition by two regionally specific antivenoms produced in Vietnam (AV. Cr. VN.) and Thailand (AV. Cr. TL.). **Results:** The patient showed good recovery, with complete normalization by day 7. SDS-PAGE profiling of the venom revealed five major enzymes, with SVSP, SVMP and PLA_2_ being the most abundant (16.7%, 40.11% and 26.11%, respectively). Antivenom inhibition tests revealed remaining casein percentages of 67.43% (AV. Cr. VN) and 59.35% (AV. Cr. TL). Blood agar assays indicated that phospholipase activity was reduced to 21.01% by AV. Cr. VN. and 23.30% by AV. Cr. TL. **Conclusions:** Our results show that the Vietnamese antivenom generated greater inhibitory activity against proteinases compared to the Thai product, underscoring the importance of using regionally specific antivenoms that are more effective against the venom profiles of locality-matched snake populations.

## 1. Introduction

Snakebite is a neglected public health crisis that results in high mortality and morbidity rates worldwide, with exacerbated severity in rural areas of tropical and subtropical regions [1]. The cause of complications and death from snakebite is due to the composition of the enzymes in snake venom. Venom is a complex biochemical mixture, comprising various biologically active components, including hydrolytic enzymes, non-enzymatic proteins or peptides, and smaller amounts of organic and inorganic molecules [2]. Together, these toxins cause local tissue damage or result in systemic toxicity, resulting in symptoms including necrosis, coagulation disorders, and even death [3]. Snake venoms contain multiple toxin families, primarily causing net cytotoxic, neurotoxic and/or hemotoxic effects. Envenomation from vipers (family Viperidae) primarily results in hemotoxic effects due to the presence of serine proteases (SVSPs), metalloproteinases (SVMPs), and phospholipase A_2_ (PLA_2_), typically leading to a pathology characterized by overt or internal bleeding and degradative effects on tissues [4].

The only currently accepted standard and efficacious treatment for snakebite envenoming is antivenom. Antivenoms are highly purified therapeutic antibody formulations derived from the plasma of animals, typically horses or sheep, that have been immunized with snake venoms [5]. These preparations consist of polyclonal antibodies, encompassing a wide variety of immunoglobulins with differing titers and affinities that target various venom components. When immunization on an antibody-producing animal is performed using snake venom sourced from a single species, the resulting product is a monovalent antivenom; however, it still comprises a polyclonal antibody mixture due to the natural immune response [6]. In contrast, polyvalent antivenoms are produced by immunizing animals with multiple venoms, or by blending several monovalent antivenoms produced as indicated previously. Typically, antibodies are isolated from the hyperimmune plasma via the protein precipitation method, and depending on the subsequent processing methods for the antibodies (e.g., enzymatic digestion), antivenom can be formulated as whole IgG molecules, F(ab’)_2_ fragments, or Fab fragments [5].

Although antivenom is considered an effective therapy for snakebite envenoming, several limitations accompany it. Due to its high cost and limited availability, antivenom may be incredibly difficult for snakebite patients to access in remote areas with inadequate healthcare services. Additionally, the effectiveness of antivenom may be significantly reduced is administration is delayed. Furthermore, only 10–20% of the antibodies in an antivenom product may target the toxin(s) of medical relevance, whereas the remaining antibodies may bind nonspecifically to host molecules, potentially decreasing venom neutralization while increasing the risk of adverse effects via hypersensitivity reactions [7]. Finally, and perhaps most importantly, variation in snake venom composition associated with geography, ontogeny, or local adaptation—both among and within species—can reduce the efficacy of antivenoms produced for geographically distant populations [8]. Therefore, it is essential to develop and produce region-specific antivenoms to ensure optimal venom neutralization efficacy and to make these products widely available and accessible.

The therapeutic efficacy of antivenoms has been demonstrated in numerous clinical cases, with favorable outcomes commonly observed following administration. However, in the case we reported highlights significant issues associated with antivenom supply, timely administration and the potential limitations of non-region-specific antivenom efficacy. This report case highlights the urgent need for *C. rhodostoma* antivenom supply across Vietnam and provides insights into the molecular mechanisms underlying variation in the efficacy of *C. rhodostoma* antivenoms. Although numerous in vivo studies have investigated venom composition and antivenom efficacy, the molecular mechanisms underlying differences in antivenom performance in clinical cases remain poorly understood. Based on the presented clinical case results, our study aims to clarify the molecular mechanisms of antivenom efficacy variation through SDS-PAGE profiling and enzyme activity assays. Our findings underscore the importance of developing regionally specific snakebite therapy and ensuring that these products are available and accessible.

## 2. Material and Methods

### 2.1. Clinical Presentation

A 22-year-old male patient with a history of bronchial asthma was bitten on the distal phalanx of the second finger of the left hand by a captive *C. rhodostoma* originating from Dak Lak Province, Vietnam. The individual brought the live snake with him to the hospital, where the species identity was verified. One hour following the bite, the patient experienced intense pain, along with swelling and bruising at the distal phalanx of the left index finger, but no bleeding or vomiting of blood occurred. The patient was admitted to the Poison Control Center at Bach Mai Hospital, Hanoi. The patient agreed and signed a consent form for the publication of their clinical information and images.

The patient was conscious at the time of admission with vital signs as follows: blood pressure 130/80 mmHg, pulse 103 beats/min, respiratory rate 20 breaths/min, SpO_2_ 99% on room air, and temperature: 37 °C. A fang puncture wound was visible on the distal phalanx of the second finger on the left hand, with swelling and mild bruising around the wound covering an area of approximately 1 cm × 2 cm. The patient’s test results are presented in detail in Table 1.

### 2.2. Venom Sample Collection

The *C. rhodostoma* specimen responsible for the envenoming was collected from the patient and housed alive in the Institute of Biology (IB) collections at the Vietnam Academy of Science and Technology (VAST) in Hanoi, Vietnam. The snake venom extraction procedure was approved by the Ethics Committee of the Institutional Ethics Committee of the Institute of Genome Research (currently the Institute of Biology), Vietnam Academy of Science and Technology (VAST), which approved the work as part of its internal research program. The specimen (Cr.2025.01) was identified as a female with a total length of 55 cm, snout–vent length of 35.5 cm, and mass of approximately 200 g. For venom collection, the snake’s mouth was rinsed with water to remove debris, and the snake was then induced to bite a parafilm membrane extended over a glass container. Once firmly latched onto the membrane, the venom glands on both sides of the jaw were gently massaged and pressed, facilitating the expression of venom into the glass container. Following collection, the venom was gently centrifuged to pellet debris and frozen at −20 °C; the sample was not subjected to lyophilization (freeze-drying). Protein concentration of the collected venom sample (SV6; snake venom 6) was 174.38 mg/mL, and was determined using the Bradford assay [10].

We tested the efficacy of two different antivenoms raised against *C. rhodostoma*: a Vietnamese product (AV. Cr. VN) obtained from Pasteur Institute in Nha Trang (Khanh Hoa Province, Vietnam, and a Thai product (AV. Cr. TL) obtained from Ho Chi Minh City, Vietnam. Protein concentrations were determined for AV. Cr. VN (74.825 mg/mL) and AV. Cr. TL (75.625 mg/mL) using the Bradford assay [10].

### 2.3. SDS−PAGE Analysis

To produce a molecular fingerprint of the protein components in the *C. rhodostoma* venom, crude SV6 venom (174.38 mg/mL) was diluted with 1X phosphate-buffered saline (PBS), pH 7.4, to a working concentration of 10 mg/mL. A 10 µg aliquot of this 10 mg/mL solution was then subjected to gel electrophoresis. We used a casein substrate to assess the proteolytic activity of snake venom proteinases, and a 1% casein solution (0.01 g/mL) prepared in phosphate-buffered saline (PBS) pH 7.4 was used as a negative control. The SV6 sample was adjusted to the same amount (0.16 µg, 0.08 µg/µL) and incubated with 1% casein (0.01 g/mL) for 10 min at 37 °C, after which the percentage activity was measured. To evaluate the inhibitory capacity of the *C. rhodostoma* antivenoms produced in Vietnam (AV. Cr. VN) and Thailand (AV. Cr. TL) against our venom sample, the SV6 sample was adjusted to the same amount (0.16 µg, 0.08 µg/µL) and incubated with each antivenom (0.16 µg, 226.2 µg/µL) for 10 min at 37 °C, after which percent activity was measured.

For gel electrophoresis, we prepared a SDS-PAGE gel using the following protocol: 4 mL of 15% resolving gel (1000 µL H_2_O, 1000 µL Tris-HCl pH 8.8 containing SDS, 2000 µL 30% bis-acrylamide, 30 µL 10% APS, and 7 µL TEMED); 1.01 mL of 4% stacking gel (610 µL H_2_O, 250 µL Tris-HCl pH 6.8, 130 µL 30% bis-acrylamide containing SDS, 12.5 µL 10% APS, and 3 µL TEMED). Each sample (9 µL) was mixed with a reducing buffer solution (0.032 M Tris-HCl, 51% glycerol, 5.1% SDS, 0.1% (*v*/*v*) saturated bromophenol blue, 4.08% β−mercaptoethanol, pH 6.8) in a 1:3 ratio and heated for 10 min at 99 °C. Samples were loaded onto the gel and run in two stages using the Mini-PROTEAN electrophoresis system (Bio-Rad, Hertfordshire, UK): stage (1) 90 volts for 15 min, and stage (2) 140 volts for 60 min. The resulting gel was stained using a buffer containing 30% Coomassie Brilliant Blue R-250 and destained for at least one hour at room temperature. Gel imaging was performed using an imaging station to capture and characterize protein band patterns.

### 2.4. Analysis of the Relative Band Intensity from the Electrophoresis Gel

The band intensity of the SDS-PAGE gel was analyzed using ImageJ Densitogram Suite version 1.54p after gel imaging software [11]. Each lane was isolated and compared, generating a densitometric plot where band intensities were represented as peaks. The area under each peak, corresponding to individual bands, was quantified using built−in measurement tools, and intensity ratios were calculated using Microsoft Excel.

### 2.5. Phospholipase Activity Assay

Agar plates were prepared as described by Suji et al. [12] with modifications. Briefly, 4% defibrinated sheep blood, 1.2% egg yolk as a source of lecithin, 10 mM CaCl_2_, and 1% agar in PBS pH 8.1 were solidified in a Petri dish, and wells of approximately 9 mm diameter were made in the gel. Samples included PBS (negative control), SV6 venom (15 µg), and mixtures of AV. Cr. VN. (15 µg) or AV. Cr. TL. (15 µg) antivenoms with SV6 venom, pre−incubated for 30 min at 37 °C, before introduction to the wells. Plates were incubated at the respective temperatures for 24 h, and the experiment was replicated three times. Hemolytic zone diameters were measured in ImageJ, and the following formula was used for the calculation of neutralization:

$$\mathrm{Percentage}\;\mathrm{of}\;\mathrm{neutralization}\;(\%)\;=\;\left(1-{\left(\frac{d_{1}}{d_{2}}\right)}^{2}\right)\times \;100$$

*d*_1_: diameter of SV6 in antivenom treatment; 

*d*_2_: diameter of SV6. 

### 2.6. Statistical Analysis

We statistically analyzed the phospholipase activity of SV6 snake venom after antivenom addition in Microsoft Office Excel 2021. We used a paired-samples *t*-test, with a significance threshold of *p* = 0.05.

## 3. Results

### 3.1. Clinical Signs and Laboratory Results Following Antivenom Administration

**Figure 1:**
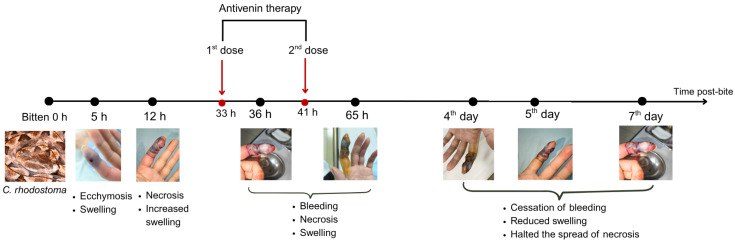
Timeline of the progression of symptoms and response to antivenom therapy for the patient bitten by *C. rhodostoma.* Outcomes were monitored for seven days following the envenomation, after which the patient was considered to have recovered fully.

**Figure 2:**
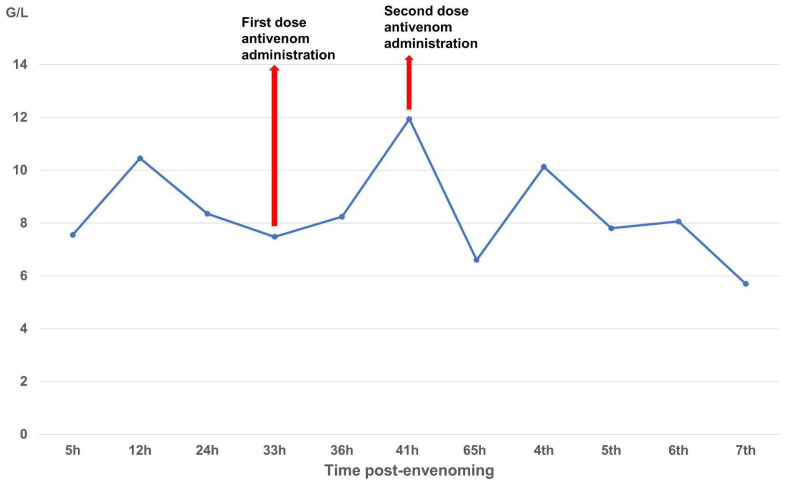
Time-course of white blood cell (G/L) changes over the seven-day monitoring period.

**Figure 3:**
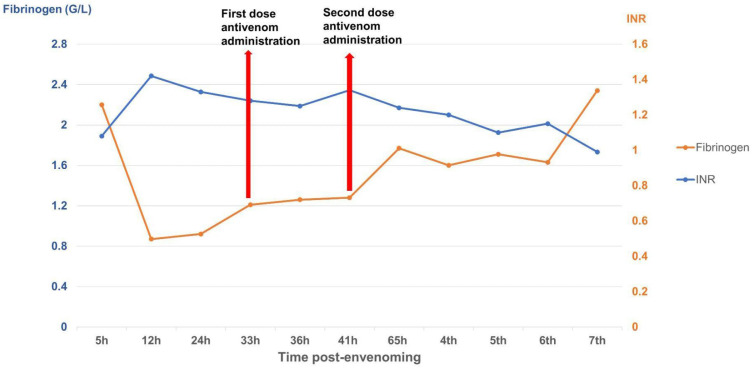
Time-course of PT-INR and fibrinogen (G/L) changes over the seven-day monitoring period.

The patient received two doses of Thai antivenom at 33 and 39 h post-bite. We observed clinical signs (Figure 1) and laboratory results, specifically focusing on WBC (Figure 2), PT-INR and Fibrinogen (Figure 3), consistent with *C. rhodostoma* envenomation. The initial WBC was 7.55 G/L, PT-INR was 1.08 and Fibrinogen was 2.20 G/L. These values subsequently changed before antivenom administration: WBC increased to 10.45 G/L, PT-INR increased 1.42 and Fibrinogen fell to 0.87 G/L. Following the first dose of antivenom, WBC decreased to 8.24 G/L, PT-INR decreased to 1.25, and Fibrinogen increased to 1.28 G/L. Minor bleeding was present at the bite site, and joint pain in the left hand significantly reduced (VAS 4-5/10), with movement remaining restricted. Following the second dose, WBC decreased from 11.94 to 7.8, PT-INR from 1.34 to 1.20, and Fibrinogen from 1.77 to 1.71 G/L. No late or additional reactions occurred from Day 4 to Day 7 post-envenomation, and swelling of the left hand continued to improve, local necrosis halted (VAS 3/10), and pain in the small/medium finger joints resolved entirely. 

### 3.2. Snake Venom Toxin Composition

**Figure 4:**
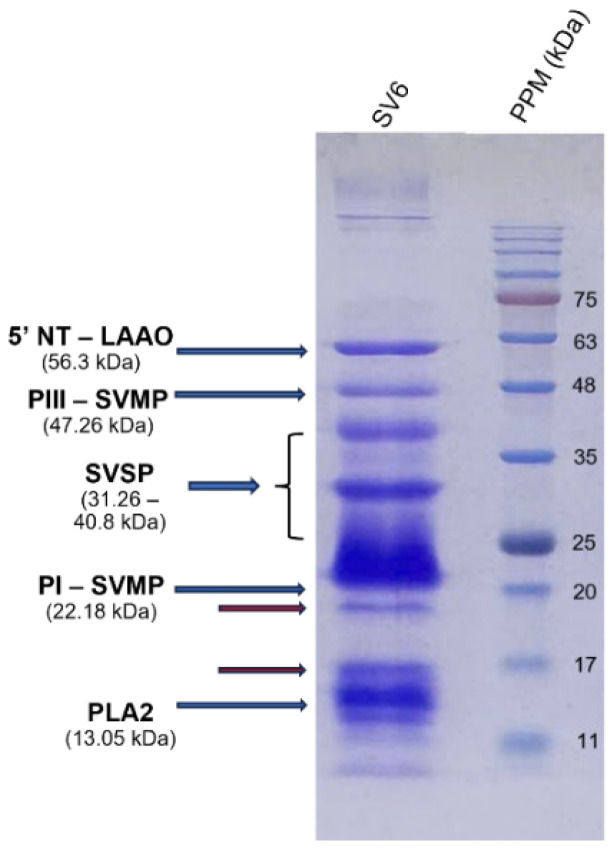
15% SDS-PAGE protein profile of SV6 *C. rhodostoma* snake venom under reducing conditions, with major toxin families labelled (SV6 = snake venom sample, PPM = protein molecular standard marker).

Gel electrophoresis (Figure 4) generated the protein band profile of the SV6 snake venom, with all bands having molecular weights below 63 kDa under reducing conditions. Five major protein bands in the venom were identified based on molecular weight, following Adisakwattana et al. [13], corresponding to: PLA_2_ (13.05 kDa), PI-SVMP (22.18 kDa), SVSP (31.26–40.8 kDa), PIII-SVMP (47.26 kDa), and 5′NT-LAAO (56.3 kDa). 

**Figure 5:**
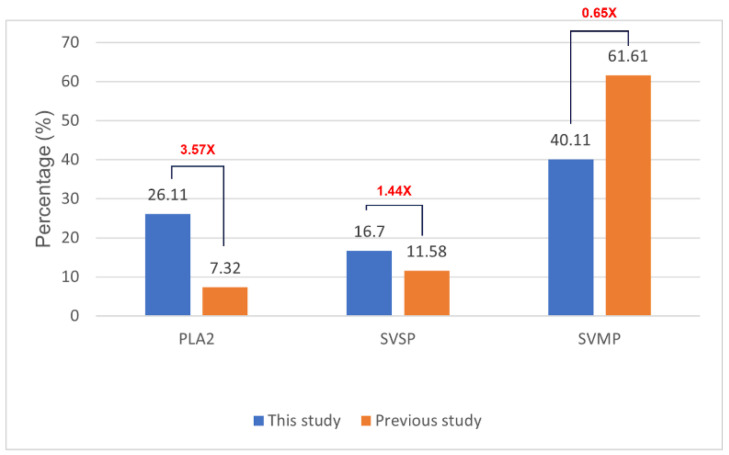
Comparison of venom protein relative abundance between *C. rhodostoma* venom analyzed in this study (SV6) and previously reported values for *C. rhodostoma* from Surat Thani Province, Thailand [13]. The red number represents the variance between the values.

Figure 5 shows the relative abundance of three major enzyme families in our *C. rhodostoma* venom sample. Our findings indicated notable differences from individuals of the same species analyzed by Adisakwattana et al. [13], which included PLA_2_ accounting for 26.11% of the venom proteome—approximately 3.57 times higher than the reported global average of 7.32%. Similarly, SVSPs comprised 16.7% of the venom proteome of SV6, approximately 1.44 times greater than the reported average of 11.58%. In contrast, SVMP abundance was significantly lower at 40.11% of the venom proteome, representing a 1.53-fold decrease compared to the global average of 61.31%. The differences in the abundances of these toxins indicate considerable variation in proportions of lethal elements among sampled individuals.

### 3.3. Neutralizing Effects of Antivenoms

**Figure 6:**
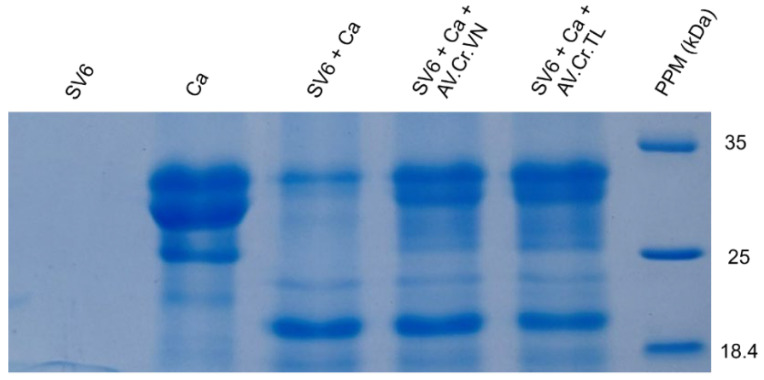
The proteolytic activity of snake venom proteinase on the casein substrate (SV6 + Ca) and the neutralized potential of the Vietnamese (SV6 + Ca + AV. Cr. VN) and Thai (SV6 + Ca + AV. Cr. TL) antivenoms, with venom only (SV6) and casein substrate only (Ca) controls indicated. Abbreviations: SV6 = snake venom sample, Ca = casein substrate, AV. Cr. VN = Vietnamese *C. rhodostoma* antivenom, AV. Cr. TL = Thai *C. rhodostoma* antivenom; PPM = protein molecular standard marker.

**Figure 7:**
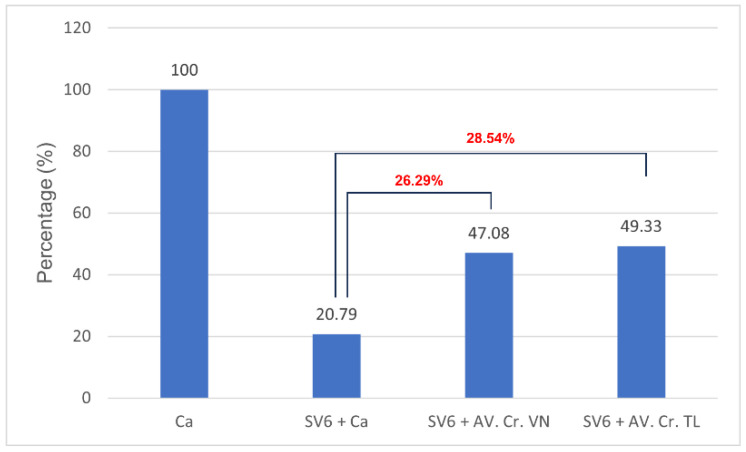
The relative inhibition of casein-degrading activity by *C. rhodostoma* proteases produced by Vietnamese (SV6 + AV. Cr. VN) and Thai (SV6 + AV. Cr. TL) antivenoms, compared to substrate-only (Ca) and venom-only reactions (SV6 + Ca). Abbreviations: SV6 = snake venom sample, Ca = casein substrate, AV. Cr. VN = Vietnamese *C. rhodostoma* antivenom, AV. Cr. TL = Thai *C. rhodostoma* antivenom. The red number represents the variance between the values.

Both the Thai (AV. Cr. TL.) and Vietnamese (AV. Cr. VN.) antivenoms had neutralizing effects on the casein-degrading activity of SV6 *C. rhodostoma* venom (Figure 6). Initially, casein (0.01 g/mL) showed three bands, with the largest occupying a position at 35 kDa, but when SV6 venom was added, it degraded casein into smaller bands. Quantitative analysis presented in Figure 7 reveals a clear, significant difference in the remaining casein content across reactions, with a reduction in all treatments. When only SV6 venom and casein substrate were present in the reaction, the enzymatic activity of the venom degraded casein significantly, with the remaining band intensity reduced to 29.03%. However, upon the addition of the antivenom—either Vietnamese or Thai—the degradation of casein was dampened substantially. The Vietnamese antivenom (AV. Cr. Vn.) increased the intensity of the casein band to 47.08%, corresponding to a 26.29% increase in intensity, while the Thai antivenom (AV. Cr. TL.) increased the intensity of the casein band to 49.33%, corresponding to a 28.54% increase in intensity. Both products were similarly efficacious in dampening the casein-degrading activity of the SV6 venom sample originating from a Vietnamese snake.

**Figure 8:**
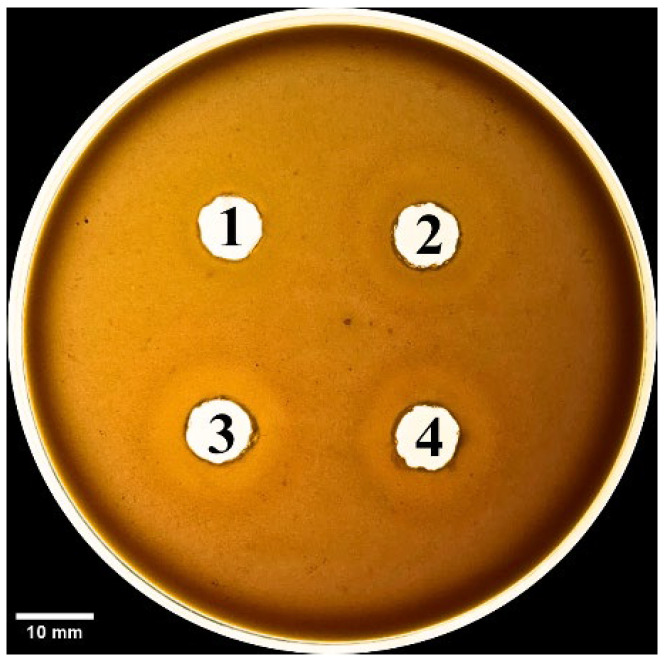
Phospholipid activity on blood agar plate, with bright zones around wells showing red blood cell lysis. Samples wells contain (1) negative control (PBS 1X, pH = 7.4), (2) SV6 venom sample (15 µg), (3) SV6 venom sample (15 µg) + Vietnamese antivenom (AV. Cr. VN.; 15 µg), and (4) SV6 venom sample (15 µg) + Thai antivenom (AV. Cr. TL.; 15 µg). Abbreviations: SV6 = snake venom sample, AV. Cr. VN = Vietnamese *C. rhodostoma* antivenom, AV. Cr. TL = Thai *C. rhodostoma* antivenom.

We also evaluated the phospholipase activity of the SV6 snake venom through indirect hemolysis of blood agar, with egg yolk lecithin used as the phospholipid source. After 18 h of incubation at 37 °C, the venom caused indirect red blood cell lysis, forming visible brighter zones around the sample wells (Figure 8). Phospholipase activity was significantly reduced to 21.01% with the addition of Vietnamese antivenom (AV. Cr. VN.) and to 23.30% with the addition of Thai antivenom (AV. Cr. VN.) (Table 2). Surprisingly, the two antivenoms showed no significant difference in their ability to neutralize venom-induced hemolysis, indicating similar inhibition of phospholipase activity for both products. 

## 4. Discussion

This clinical case presented here was novel, as it involved a patient bitten by a Malayan pit viper (*C. rhodostoma*) under unusual circumstances. Epidemiologically, bites from this species are primarily associated with wild snakes from within the native geographic distribution in Southern Vietnam, as the range of this species does not extend into Northern Vietnam [14]. The case presented herein, received at the Poison Control Center, Bach Mai Hospital, Hanoi, is thus considered anomalous, and consequently, species-specific antivenom was not available for immediate administration. The envenomation occurred because the patient (a veterinarian) owned the captive snake and was bitten on the distal phalanx of the second finger of the left hand during a venom extraction. The patient brought the snake to the Poison Control Center, facilitating accurate species verification and therefore allowing sourcing of appropriate antivenom. The bite could additionally be confirmed by one fang mark puncture visible on the individual’s left second finger, corroborating the need for monitoring of envenomation symptoms. When species responsible for snakebites cannot be identified, diagnoses become reliant on bite wound characteristics, local symptoms (including the presence of one fang mark, swelling, blistering, or necrosis), and laboratory tests. These tests may include prolonged clotting times (Lee White, PT, aPTT), platelet counts (<20.000/mm^3^), decreased (sometimes undetectable) fibrinogen levels, elevated D-dimer levels, and non-clotting blood [15]. ELISA can also be used for species identification and can additionally quantify venom doses based on venom antigens [16]. However, these diagnostic methods (i.e., ELISA) have significant limitations, such as conserved signals across different venomous snake species, and the risk of cross-reactivity with venoms from closely related snake species producing incorrect identifications. The limit detection of ELISA for venoms and toxins ranges from 0.1 to 20 µg/L, and it requires a long antibody incubation period [17,18], Researchers are developing rapid detection methods to overcome these shortcomings, such as using bite swabs that can generate effective results through PCR testing [19,20].

The case described here met conditions for antivenom administration due to local necrosis and coagulopathy immediately evident in paraclinical results. However, antivenoms were unavailable locally and required transport from Ho Chi Minh City to Hanoi, resulting in major delays with the first dosage being administered 33 h and the second dosage 39 h post-bite. Antivenom should be administered as soon as possible following a snakebite, ideally within 4 h as per manufacturer guidelines, though it can still be effective for as long as 24 h post-bite. Considering the delayed administration of antivenom in our case, we observed rapid improvement in the patient’s symptoms and laboratory results. Three important parameters for evaluating the impact of snake envenomation and antivenom on the immune system include WBC (normal range 4–10 G/L) for inflammation, INR (normal range 0.8–1.2), and fibrinogen (normal range 2–4 G/L) for coagulation. After the envenomation, the venom caused tissue damage and triggered an inflammatory response, resulting in WBC increasing to 10.45 G/L by day 2 post-bite. The venom also caused clotting disorders and bleeding, evidenced by the depletion of the clotting factor fibrinogen to 0.87 G/L and extending the coagulation INR to 1.42. These parameters tended to increase slightly for fibrinogen and decrease for INR and WBC to normal levels as the patient was treated with supportive medicines, including analgesics and antibiotics. Following antivenom administrations, these parameters gradually improved to normal levels in the subsequent days of treatment. Interestingly, WBC initially increased above normal to 11.94 G/L after antivenom administrations, most likely due to an immune response to the antivenom acting as an antigen; however, these levels rapidly normalized (<10 G/L), indicating no adverse reactions. Our clinical results demonstrate that the timing of antivenom administration is crucial for effectively reducing venom toxicity, but support the use of antivenom even when administration is delayed. 

The effectiveness of antivenom largely depends on the toxin composition profile of the respective snake venom. To elucidate the molecular mechanisms of antivenom activity, we constructed a protein profile of the *C. rhodostoma* responsible for the envenomation described in this case. We provided important insights into the molecular mechanisms that caused clinical symptoms, as well as evaluated the efficacy of two different antivenom products in neutralizing toxin activity. We identified three major enzyme families, including SVMPs, SVSP, and PLA_2_, which are related to the clinical presentation of *C. rhodostoma* envenomed patients. Specifically, SVMPs contribute to extracellular matrix protein degradation, leading to tissue breakdown and necrosis [21]. SVSPs are thrombin-like enzymes, cleaving fibrinogen and causing coagulation disorders [22]. The proteolytic activities of both SVSPs and SVMPs can activate inflammatory responses and cause hypotension [23,24]. Meanwhile, PLA_2_ hydrolyzes the phospholipids in red blood cell membranes, resulting in hemorrhage and swelling. The severity of the effects induced by these toxic components is associated with their respective relative abundances in snake venoms. We found significant differences in proportions of toxin components of SV6 venom when compared to the previous reports of *C. rhodostoma* venom from Surat Thani, Thailand, by Adisakwattana et al. [13]. These differences may be explained by geographic variation, diet, age, sex, and physiological differences among individuals. Additional research on geographic venom variation represents a vital first step in improving and progressing the effectiveness of species-specific antivenom products. 

We also evaluated the efficacy of antivenoms at the molecular level. In our case, the patient was treated with antivenom produced in Thailand (AV. Cr. TL), which was transported from Ho Chi Minh to Hanoi. Although *C. rhodostoma* antivenom in Vietnam (AV. Cr. VN) has been researched and tested, it remains uncommercialized and unavailable. Our neutralization experiments showed that both Vietnamese (AV. Cr. VN) and Thai (AV. Cr. TL) antivenoms had equivocal efficacies in inhibiting proteases from our sampled Vietnamese individual of *C. rhodostoma*. Evidencing this, when venom was combined with each antivenom on the 15% SDS-PAGE, the number and intensity of casein protein bands were greater than those of antivenom-free treatments. The zones of phospholipid degradation (diameter, measured in mm) on a blood agar plate were reduced in treatments with either of the two antivenom products compared to treatments lacking antivenom. Furthermore, quantitative analyses also demonstrated that AV. Cr. VN (Vietnam) was more efficacious in neutralizing proteinase activity than AV. Cr. TL (Thailand), further emphasizing the importance of using regionally derived antivenoms to optimize neutralization of activities.

Our case report highlights the reliance on antivenoms produced in other countries and the limitations imposed by domestic transportation and distribution of antivenom in Vietnam, inhibiting the rapid administration of antivenom and reducing the effectiveness of treatment. WHO data on global antivenom production reveal heterogeneity among manufacturers in terms of production scale and antivenom characteristics [1]. Some manufacturers produce antivenoms only for their own country, while others distribute products regionally or globally [21]. Furthermore, geographical variation in snake venom composition can lead to reduced specificity and efficacy when using antivenoms produced in neighboring countries [22]. With these challenges in mind, promoting research and establishing industrial-scale production of specific antivenoms for local Vietnamese snake species is a promising direction for future efforts. Simultaneously, exploring alternative therapies to traditional antivenoms is a crucial step, especially when antivenoms are unavailable or ineffective in abating clinical symptoms. This proposed approach could shorten waiting times, enhance treatment efficacy, and reduce the burden of severe snakebite complications in Vietnam.

This study has several limitations, including reporting a single case and presenting an in vitro scope. The main geographical distribution of this snake species is in the Southern provinces, and this was the first case reported in the Northern area. The clinical presentation, however, was consistent with the multiple case study in Malaysia, with most of the abnormalities occurring after 6 h post-bite [25]. For further investigation, in vivo studies need to be carried out using animal models.

## 5. Conclusions

Malayan pit viper bites are rare in northern Vietnam, but the case presented here displayed characteristic clinical signs and coagulopathy and showed a clear improvement in response to antivenom administration. Although geographic variation in snake venom might have inhibited the effectiveness of antivenom used in the treatment of this envenomation, the clinical outcome following antivenom treatment was generally favorable, and the patient made a full recovery. However, the regionally mismatched Thai antivenom (AV. CR. TL) has been shown to lack efficacy in immunological binding and neutralization of the Vietnamese *C. rhodostoma* venom sample tested herein [26]. Coupled with our in vitro study, we emphasize that antivenom therapy should consider geographic variation in snake venoms and urge clinicians to consider the geographic and taxonomic coverage of the antivenom products that they have access to.

## Figures and Tables

**Table 1:** Laboratory results at admission.

Test Description	Result	Unit	Normal Range
*Complete blood count* (*CBC*)			
RBC	5.33	T/L	4.37–5.69
Hb	154	G/L	136–175
PLT	232	G/L	150–400
WBC	7.55	G/L	4.0–10.0
*Coagulation*			
PT (s)	14.1	s	
PT	89	%	70–140
INR	1.08		0.8–1.2
APTTs	29.6	s	
APTT ratio	1.00		0.8–1.2
Fibrinogen	2.2	G/L	2–4
D-Dimer	5.146	mmg/L LEU	
Coagulation test	positive		
*Urine biochemistry*			
RBC	0	G/L	negative
WBC	0	G/L	negative
Protein	0	G/L	negative
*Blood biochemistry*			
Urea	3.9	mmol/L	2.76–8.07
Creatinine	76	µmol/L	72–127
Glucose	4.9	mmol/L	4.1–5.9
GOT	16	U/L	<50
GPT	8	U/L	<50
Na	140	mmol/L	136–146
K	3.7	mmol/L	3.4–4.5
Cl	102	mmol/L	101–109
*ROTEM*			
Intrinsic pathway			
CT	215	s	100–240
CFT	199	s	30–110
α	57	°	70–53
A5	26	mm	>35
MCF	45	mm	50–72
ML	* 10	%	<15
LI60	90	%	>85
Extrinsic pathway			
CT	90	s	38–79
CFT	224	s	34–159
α	54	°	63–83
A5	45	mm	50–72
MCF	45	mm	9–25
ML	* 9	%	<15
LI60	92	%	>85
Platelet inhibition			
CT	83	s	38–79
A5	5	mm	7–9
MCF	5	mm	9–25
ML	* 0	%	<15
LI60	100	%	>85
Bedside clotting [9]	Positive		

*: the data are statistically significant. *C. rhodostoma* antivenom was not available at the hospital, and consequently the patient received supportive treatments including analgesics (Paracetamol 1000 mg x 1 vial IV and Morphine 10 mg/mL x ½ ampoule IM); antibiotics (Ciprofloxacin 200 mg x 4 vials IV and Clindamycin 300 mg x 3 ampoules IV); Tranexamic acid (250 mg/5 mL x 4 ampoules IV); and Methylprednisolone (40 mg x 2 vials IV) before antivenom administration occurred. At 33 h post-bite, the patient received 1 vial of Thai *C. rhodostoma* antivenom (Thai Red Cross, Lot No. 002-00-21), diluted in 250 mL of 0.9% NaCl, administered via IV at a rate of 120 mL/h, following a 15 min negative intradermal skin test. The second vial of Thai antivenom was administered 6 h later following the same protocol.

**Table 2:** Diameter of hemolysis zone (mm) and percentage of hemolysis neutralization (%) produced by Vietnamese and Thai antivenoms. Abbreviations: SV6 = snake venom sample, AV. Cr. VN = Vietnamese *C. rhodostoma* antivenom, AV. Cr. TL = Thai *C. rhodostoma* antivenom.

Sample	Diameter of Hemolysis (mm)	Percentage of Neutralization (%)
SV6	19.93 ± 0.88	0
SV6 + AV. Cr. VN	17.71 ± 0.37 *	21.01
SV6 + AV. Cr. TL	15.45 ± 1.13 *	23.30

*: the data are statistically significant. Values are expressed as means ± standard error of the mean (*n* = 3), and analysis was performed with paired-samples *t*-test compared to SV6 with a significance threshold of *p* = 0.05.

## Data Availability

The data presented in this study are available on request from the corresponding author.
